# Spinal Cord Injury and Autonomic Dysreflexia: A Case Report on an Overlooked Complication of Spinal Cord Injury

**DOI:** 10.7759/cureus.30259

**Published:** 2022-10-13

**Authors:** Ahmad H Alwashmi

**Affiliations:** 1 Department of Orthopedic Surgery, College of Medicine, Qassim University, Buraydah, SAU

**Keywords:** disability, physical medicine and rehabilitation, spasticity, spinal cord injury, autonomic dysreflexia

## Abstract

Autonomic dysreflexia (AD) is a life-threatening condition that affects patients with spinal cord injuries (SCI) at the sixth thoracic vertebrae (T6) and above due to a noxious stimulus below the level of spinal cord injury. This is a case report of a 48-year-old man with a history of paraplegia T1 (American Spinal Injury Association Impairment Scale - ASIA A) spinal cord injury due to a road traffic accident 16 years ago who presented with recurrent episodes of hypertension, sweating, bradycardia, and hypothermia. Previous hospitalizations suggested that his symptoms were caused by sepsis from a urinary tract infection; however, further assessment revealed that his symptoms were consistent with untreated and undiagnosed autonomic dysreflexia. This case report provides an overview of AD, including its distinctive presentation, etiology, pathophysiology, and management. Autonomic dysreflexia can be a life-threatening condition associated with spinal cord injury patients at the T6 level and above due to various noxious stimuli below the neurological level of injury. Bladder distension appears to be the trigger in most of the cases reported. AD can be easily missed by medical staff unfamiliar with this condition. Patient and healthcare provider education and a thorough evaluation are essential for diagnosis and management.

## Introduction

Autonomic dysreflexia (AD) is a condition that occurs shortly after spinal cord injury (SCI), usually when the injury occurs at or above the thoracic (T6) level and is unlikely to manifest if the injury occurs below T10; most patients report having AD after a period of spinal shock when reflexes are restored, usually after the first year post-injury; however, there have been a few cases reported as early as the fourth day post-injury. Additionally, autonomic dysreflexia can occur more than once per day in susceptible individuals, and patients with higher-level spinal cord injuries (cervical, high thoracic T6 and above, and complete spinal cord injuries) are up to 90% more likely to develop AD [1-2].

In Saudi Arabia, a study of Saudi male SCI patients found that 43.9% had a cervical injury, 40.4% had a thoracic injury, and 3.5% had a lumbar injury; this increases the odds of encountering AD with SCI patients [3]. It is defined as a syndrome in spinal cord injury patients involving a sudden, excessive, reflexive rise in blood pressure to stimuli below the neurological level of injury triggered by visceral or sensory stimuli resulting in unopposed spinal cord medicated sympathetic reflex, usually urinary tract infection, bladder or bowel distension. Also, it can be associated with acute abdomen conditions such as appendicitis, cholecystitis, or even undetected fractures and pressure injuries [4]. It is usually accompanied by a severe headache, bradycardia, and flushing of the face, along with pallor, cold skin, and sweating (above the neurological level). It is important to note that this can be a potentially fatal condition that can occur in more than half of potentially susceptible individuals; however, it is usually easy to manage with prompt detection and relatively simple corrective action by caregivers [5]. Many serious complications following AD have been reported, including, stroke, seizure, myocardial infarction, and sudden death [6]. Patients with a traumatic spinal injury who have AD have significantly higher mortality rates than similarly injured individuals who do not have the disorder [7].

The initial presenting complaint is usually a headache, throbbing in character. Individuals with SCI higher than T6 should regularly have their blood pressure measured. If hypertension is detected, it is likely they have AD [8-9]. Unfortunately, many emergency room staff including nurses, and physical therapists are not familiar with AD, and are unable to recognize or manage it quickly [10-11].

## Case presentation

A 48-year-old male with spinal cord injury and subsequent paraplegia, T1 ASIA A (American Spinal Injury Association impairment scale) [12] due to a road traffic accident, neurogenic bladder dysfunction, and frequent urinary tract infections presented to his family care physician with a three-day history of severe sweating, throbbing headache, elevated blood pressure, and increasing lower limbs spasticity. On further history taking and review of his chart, there were numerous clinic visits, emergency department visits, and hospital admissions as well with similar symptomatology. He was managed and received multiple antibiotics for his urinary tract infections without further investigation or evaluation for his extreme diaphoresis, headache, and hypertension. He was also trialed on multiple antihypertensive medications with no improvement, and several medications caused side effects such as dizziness and constipation. As for his bladder management, he displayed awareness of clean intermittent catheterization (CIC) as he had received proper education on this procedure. However, he refused self-catheterization due to fear of urethral trauma and managed his bladder by Credé's maneuver by applying suprapubic pressure to facilitate urination. On his recent admission, he was complaining of excessive sweating, headache, anxiety, and difficulties in urinating. He denied any change in urine color or malodor.

On examination, he was alert, oriented to time, place, and person, with an average body build, wearing tight socks, tremulous, and diaphoretic from the nipple line and above, necessitating the use of a towel to wipe excess perspiration from his face

**Figure 1:**
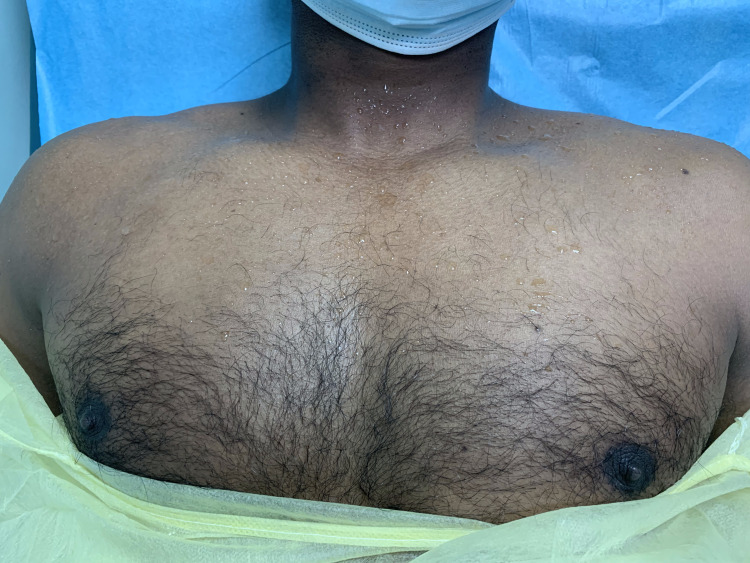
Excessive perspiration

Figure 1 shows (with patient permission) excess perspiration on the patient's chest during an autonomic dysreflexia (AD) episode. 

Vital signs revealed high blood pressure levels of 187/110 mmHg, a low oral temperature of 34.6 Celsius (94.3 Fahrenheit), and a bradycardia heart rate of 46 beats per minute. There were no pressure injuries or changes in skin color. Neurological examinations showed sensorimotor deficits below T1. Muscle power testing showed 0 out of 5 below T1 myotome with increased spasticity of 3 out of 4 on the modified Ashworth scale on major flexor muscles of the lower extremities.

The post-residual volume of the urinary bladder showed an amount of 236 ml urine analysis showed clear yellow (light) color PH 5.6 negative nitrate, and leukocyte esterase, protein<150 mg/d, red blood cells (RBCs) <2/hpf, white blood cells (WBCs) 4/hpf, and 4 squamous epithelial cells.

The patient was reassured, his tight socks and clothes were removed, and was dressed in a hospital gown. The nursing staff were instructed to sit the patient up. Intravenous normal saline and empirical antibiotic were started as well, and a urine culture was sent along with clean intermittent catheterization as he refused an indwelling urine catheter due to previous complications. Within a few minutes of close observation, his blood pressure dropped to 149/88 mmHg, his oral body temperature was 37.2 Celsius (98.96 Fahrenheit), and his heart rate was 58 beats per minute. On the next day, his blood culture remained with no growth, and his urine culture grew Klebsiella pneumoniae. The infectious disease team was consulted, and proper antibiotics were started. On day two, his blood pressure normalized, and the excessive perspiration and headache resolved. Before his discharge, the patient was educated about AD, including causes and measures to follow to prevent future episodes of AD as it is considered a life-threatening emergency. We also provided him with a card that summarizes AD and steps for management to present to unfamiliar healthcare providers and counseled him about the possibility of suprapubic catheter insertion as he exhibited fear of using clean intermittent catheterization; urology consultation was initiated for this matter and a requisition for a urodynamic study as well.

## Discussion

This case study highlights the significance of promptly diagnosing and treating patients with SCI and AD. This patient had visited his primary physician for undiagnosed clinical manifestations that had been indicative of AD. Based on recent admission, cystitis, tight stockings, and noxious irritation from chronic bladder overdistention are three potential causes of AD.

Understanding the pathophysiology of AD is essential to management. In a healthy individual, blood pressure is controlled by the autonomic nervous system's coordination of the parasympathetic and sympathetic nervous systems. In the case of patients with spinal cord injuries (as in the present case), any noxious stimuli below the level of SCI, such as bladder overdistention or infection, elicits an exaggerated sympathetic outflow from the spinal cord with a lack of compensatory corrective descending parasympathetic stimulation, resulting in vasoconstriction and systemic hypertension similar to our patient. Normally, the parasympathetic nervous system promotes splanchnic vascular bed vasodilation in order to lower systemic blood pressure. In the event of SCI like our patient, the only parasympathetic tone present with AD is baroreceptor-mediated activation of the vagus nerve in response to systemic hypertension. This eventually results in bradycardia and vasodilatation above the level of SCI, as manifested by the patient's heart rate, but is not enough to stabilize his hypertension. Hypothermia, or low body temperature, is another important feature of AD secondary to thermoregulation dysfunction, loss of sympathetic control of temperature, and sweat regulation. A case of hypothermia associated with AD has previously been described in the literature, with the hypothesis that excessive perspiration, along with thermoregulation dysfunction, contributes to hypothermia; however, there is limited evidence available regarding the implications of AD on thermoregulatory dysfunction [13].

Normal functional bladder capacity in adults ranges between 300-400 mL and bladder storage is predominantly a sympathetic response whereas bladder emptying is facilitated by a parasympathetic response [14]. Due to various noxious stimuli, patients with SCI may experience abnormal sympathetic stimulation of the bladder. In fact, bladder distension appears to be the trigger in 75% to 85% of cases of AD [15]. Despite utilizing the Credé maneuver, our patient had a post-void residual of 236 mL owing to increased sympathetic stimulation. For patients with SCI, close monitoring of bladder health is essential, and urodynamic studies should be carried out to determine the type of neurogenic bladder dysfunction and whether to start appropriate medications such as anticholinergic medications, also to avoid complications such as AD, incontinence, renal impairment, and urinary tract infection [16].

As shown in Table 1, there are few cases of AD reported. Most of these cases have common symptoms of AD, such as headache, high blood pressure, and excessive perspiration. Patients with spinal cord tumors and non-traumatic spinal cord injuries, such as multiple sclerosis, are less likely to develop AD, and yet there have been three case reports of intramedullary astrocytoma of the spinal cord, multiple sclerosis, and cervical spinal cord gliofibroma associated with AD [17].

**Table 1:** Previous case reports of AD-associated spinal cord injury patients

Title	Age	Neurogenic status level	Presentation	Authors and reference
Spinal cord injury and autonomic dysreflexia: a case report	51/M	Complete C6	Headache, severe sweating, BP: 170/100 mmHg	Bhatt et al [18]
Autonomic dysreflexia-induced reversible posterior leukoencephalopathy syndrome in patients with spinal cord injury	58/M	Complete C5	Headache, BP:160/80 mmHg	Joa et al. [19]
Autonomic dysreflexia associated with Charcot spine following spinal cord injury: a case report and literature review	50/M	Complete C8	BP: 250/150 mmHg, headaches, excessive sweating	Morita et al [20]
Autonomic dysreflexia after hip fractures managed by regional anesthesia: a case report	57/M	Complete C5	Flushing, profuse diaphoresis, anxiety, BP 178/111 mmHg	Huynh et al [21]
Seizures: a rare presentation of autonomic dysreflexia in a young adult with complete spinal cord injury	18/M	Complete C4	Seizure, headache, and facial flushing BP180/120 mm	Hartman et al [22]
Recurrent stroke in a patient with spinal cord injury due to autonomic dysreflexia: a case report	56/M	Complete C5	Severe perspiration, altered mental state, headache, and severe hypertension	Broecke et al [23]

As previously discussed, early detection and intervention of AD are crucial to avoid serious complications [6]. A thorough assessment is essential to determine the patient’s clinical history, establish their level of risk, and identify the likely cause of autonomic dysreflexia. The management of AD involves both pharmacological, and non-pharmacological interventions.

Table 2 shows step-by-step interventions for AD management.

**Table 2:** Step-by-step interventions for AD management

1- Sit the patient upright
2- Loosen any tight clothing or socks
3- Monitor the blood pressure every 2 to 5 minutes during the episode
4- If no indwelling catheter is present, perform an intermittent catheterization
5- If an indwelling catheter is present, check it for obstructions and irrigate the catheter
6- If symptoms are still present and systolic blood pressure is 150 mmHg or greater, treat the blood pressure pharmacologically
7- If symptoms are still present and systolic blood pressure is less than 150 mm Hg, manually evacuate the bowel.
8- If symptoms persist, search for other causes, i.e., ingrowing toenails, pressure ulcers, fractures, etc.

In many cases, removing the noxious stimulus below the level of SCI, such as tight socks, and/or draining an overdistended urinary bladder is sufficient to eliminate AD. Sitting the patient up is an important initial step in management. This will help lower blood pressure orthotactically by inducing the pooling of blood in the abdominal, and lower extremity vasculature, as we encountered with our patient. Close observation of the patient’s vitals every two to three minutes during an AD episode and observing for recurrence for the next two to three hours. Rapidly acting medications such as nifedipine 10mg sublingual or chewed, or glyceryl trinitrate spray (one to two sprays) are indicated as first-line treatment if systolic blood pressure remains above 150 mmHg, for two to three minutes and after exhausting the non-pharmacological measures [5]. Patient education is also an important component of the long-term management of AD, enabling the patient to act promptly to avoid complications. Furthermore, patients with recurrent AD should be taught self-management techniques such as bladder/bowel emptying and should be provided with an alert card to present when necessary, as we did with our patient. This will enable healthcare providers unfamiliar with this condition to act rapidly and effectively.

## Conclusions

Autonomic dysreflexia (AD) can be a life-threatening condition associated with spinal cord injury patients at T6 level and above due to various noxious stimuli below the neurological level of injury. Bladder distension appears to be the trigger in most of the cases reported. AD can be easily missed by medical staff unfamiliar with this condition. Patient and healthcare provider education and a thorough evaluation are essential for diagnosis and management.
